# ASQ3 and/or the Bayley-III to support clinicians' decision making

**DOI:** 10.1371/journal.pone.0170171

**Published:** 2017-02-02

**Authors:** Robin Mackin, Nadya Ben Fadel, Jana Feberova, Louise Murray, Asha Nair, Sally Kuehn, Nick Barrowman, Thierry Daboval

**Affiliations:** 1 University of Ottawa, Ottawa, Ontario, Canada; 2 Children’s Hospital of Eastern Ontario, Department Pediatrics, The Ottawa Hospital, Department of Obstetrics and Gynecology, Ottawa, Ontario, Canada; 3 Children’s Hospital of Eastern Ontario Research Institute, Clinical Research Unit, Ottawa, Ontario, Canada; University Children's Hospital Tuebingen, GERMANY

## Abstract

**Background:**

Appropriate tools are essential to support a clinician’s decision to refer very preterm infants to developmental resources. Streamlining the use of developmental assessment or screening tools to make clinical decisions offers an alternative methodology to help to choose the most effective way to assess this very high-risk population.

**Objective:**

To examine the influence of the Ages and Stages Questionnaire-3^rd^ edition (ASQ3) and the Bayley Scales of Infant Development-3^rd^ edition (Bayley-III) scores within a clinically-based decision-making process.

**Methods:**

This retrospective cohort study includes children born at less than 29 weeks gestation who had completed both psychologist-administered Bayley-III and physician-observed ASQ3 assessments at 18 months corrected age. Theoretical referral decisions (TRDs) based on each assessment results were formulated, using cut-off scores between the lower first and second standard deviation values and below the lower second standard deviation values. TRDs to refer to developmental resources were evaluated in light of the multidisciplinary team’s actual final integrated decisions (FID).

**Results:**

Complete data was available for 67 children. The ASQ3 and the Bayley-III had similar predictive value for the FID, with comparable kappa values. Comparisons of the physicians’ and psychologists’ TRDs with the FIDs demonstrated that the ASQ3 in conjunction with the medical and socio-familial findings predicted 93% of referral decisions.

**Conclusion:**

Taking into consideration potential methodological biases, the results suggest that either ASQ3 or Bayley-III, along with socio-environmental, medical and neurological assessment, are sufficient to guide the majority of clinicians’ decisions regarding referral for specialty services. This retrospective study suggests that the physician-supervised ASQ3 may be sufficient to assess children who had been extremely preterm infants for referral purposes. The findings need to be confirmed in a larger, well-designed prospective study to minimize and account for potential sources of bias.

## Introduction

Timely identification of children at risk of developmental and medical complications and in need of support is important, as are accurate referrals to resources for targeted interventions to promote the best outcomes for high-risk populations [1,2]. For children born very prematurely, experts have been actively seeking to develop well-structured and cost effective neonatal follow-up (NNFU) programs, and to improve multidisciplinary team effectiveness in the context of limited financial and human resources [3].

Medical decisions to refer to developmental resources are complex, as practitioners synthesize multiple sources of qualitative clinical information along with the results of the developmental assessment tools. Opinion varies as to what constitutes appropriate indications for referral to developmental resources. One practitioner may believe that a confirmed diagnosis based on a specific developmental problem requiring intervention is necessary, whereas another may consider a referral based on scoring on developmental screening tools along with other sources of information to be sufficient for referrals to improve language, cognitive and academic skills [4]. Our multidisciplinary team strongly supports the idea that appropriate referrals be made as early as possible when delays in milestones acquisition are suspected, before a diagnosis is necessarily confirmed [5].

Instruments to screen and to identify infants who are not reaching developmental milestones appropriate for their age should be used to guide clinicians’ impressions and decisions to refer to appropriate developmental resources. Two commonly used tools are the Bayley Scales of Infant Development (Bayley), a developmental assessment, [6] and the Ages and Stages Questionnaire (ASQ), a screening tool [7].

The Bayley is widely used to report on the developmental outcomes of children born very preterm [6]. Many experts consider this tool to be the mainstay to assess children between 18 and 24 months corrected age [8], albeit with the caveat that this tool is expensive to administer. The ASQ, a parent-completed screening tool, holds some promise to assess a similar age range, rendering it a cost-effective tool to identify low-risk babies who require more in-depth assessments [7].

The validity of the ASQ to predict developmental delays following high-risk very premature birth has been compared with the Bayley with inconclusive results [9–12]. The ASQ sensitivity and specificity failed to reach levels of 70% to 80% consistently, to identify children with developmental delays [13].

Focusing solely on the concurrent validity properties of the ASQ to predict developmental delay may not be sufficient to guide clinical decisions. Concurrent validity studies do not capture how the professionals who are assessing infant development use the ASQ and the Bayley to support medical decisions to refer to developmental resources. Independently from the test scores, direct observation of the infant performing tasks proposed by the instrument or parental reporting of their infant’s performance during the assessment may influence clinicians’ decisions. We therefore hypothesized that the ASQ-3^rd^ edition (ASQ3) answered and observed by a physician with additional information of the parents when in doubt, would support majority of clinicians' decisions to refer to developmental resources, despite uncertainties regarding the accuracy to detect developmental delay compared to the Bayley-3^rd^ edition (Bayley-III). We sought to examine the influence of the ASQ3 and the Bayley-III scores within a multidisciplinary clinically-based decision-making process, in order to evaluate a cost-effective way to assess development without compromising clinical decision-making.

## Methods

### Study design and population

This retrospective cohort included children who had been born at less than 29 weeks gestation, between April 1^st^ 2009 and March 31^st^ 2011; had been seen at our regional quaternary care facility at the Children’s Hospital of Eastern Ontario (CHEO), Ottawa, Canada neonatal follow-up (NNFU) clinic for their 18 months assessment. We included children who had been extremely preterm infants, and who had been administered both an ASQ3 and Bayley-III independently at 18 months corrected gestational age; children with an incomplete ASQ3 or Bayley-III assessment leading to missing data were not included in the analysis. Neither were children not seen at their 18 months old corrected age visit as their care had been transferred to the rehabilitation center for treatment of severe handicap prior to that age, or who were being followed in another paediatric clinic as per geographical criteria. The Children’s Hospital of Eastern Ontario Research Ethics Board approved the study. Considering the retrospective design of the study consent was not required, patient records and information was anonymized and de-identified prior analysis.

### NNFU clinic workflow

In our region, infants at high risk for developmental delay are usually seen at 4, 10 and 18 months corrected age and at 4 years of age. During these visits children are assessed by a multidisciplinary team including a nurse, one of five paediatricians involved in the clinic, a physiotherapist at the 4 months corrected age visit and a psychologist at the 18 months corrected age visit.

At the 18 months corrected gestational age visit the nurse reviews the medical history, and assesses feeding, growth, behavior and socio-familial status through a structured interview. The registered psychologist reviews the nurse's assessment if already completed, reviews the socio-familial status, assesses behavior, and administers and scores the Bayley-III [6]. This tool is used to assess development of children from 1 to 42 months of age. It determines scores for cognitive, language and motor skills through standardized items and scores for social and adaptive abilities through parental questionnaires. Bayley-III provides norm-referenced composite scores for each skill area with a mean of 100 and a standard deviation of ± 15. Independent of the psychologist’s assessment, the physician reviews the nurse’s assessment before seeing the infant and the family, completes a medical assessment that includes a physical and neurological examination, and along with the parent uses the 18 months old questionnaire of the ASQ3 to score the infant’s developmental performance. The ASQ3 is a screening tool composed of 21 age-specific questionnaires covering the range 1 to 66 months of age [7]. Each questionnaire includes 30 developmental items divided into five domains: communication, gross motor, fine motor, problem solving and personal-social. Although the ASQ3 is designed to be completed by parents, the NNFU team members use the tool to guide the interview with parents and to guide the face-to-face observation of the child’s abilities to perform the milestones, such as stacking of 4 or 5 small size blocks. When a child did not perform an item or when the clinic design was not appropriate for a particular task, for example finding a familiar toy or object in another room, parents were invited to mention whether they witnessed their infant performing that ASQ3 item, and the information was included in the final score.

**Fig 1:**
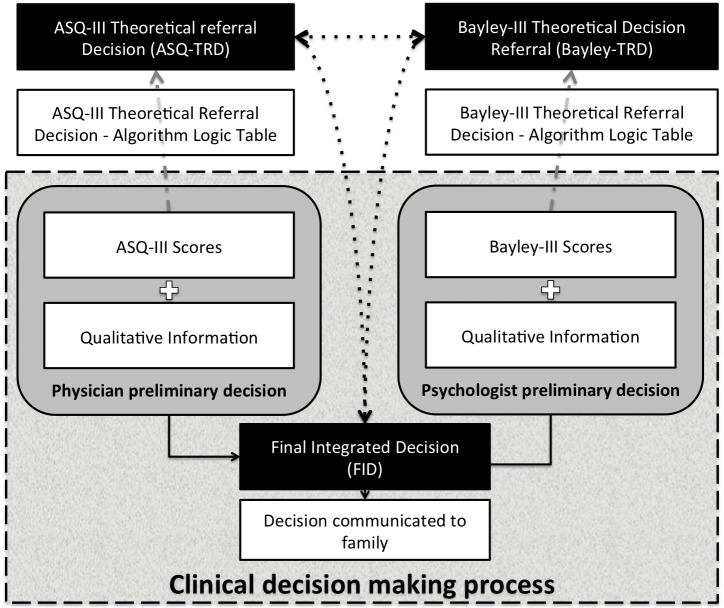
18 months old corrected gestational age decision-making process and analysis. Qualitative information includes information gathered from the medical, behavioral and socio-family environmental assessment and the neurological exam. ASQ, Ages and Stages Questionnaire; Bayley-III, Bayley Scales of Infant Development 3^rd^ edition; FID, Final integrated decision; TRD, Theoretical referral decision.

Fig 1 illustrates a typical clinical decision making process that would correspond to most of the clinical situations, as per NNFU team members agreement; however in individual cases the sequence of the different examinations and sources of information used in clinic to come to the decisions that are ultimately communicated to the family may have been adjusted. In short, the psychologist used and scored the Bayley-III and the physician scored the ASQ3 as originally designed [6,7]; both independently administered the screening or the developmental assessment tool, before communicating with one another regarding the particular child. The qualitative information that may also influence decisions with regard to developmental resources includes the socio-familial status, and the behavioral, medical, physical and neurological assessments. The combination of the ASQ3 scores and the qualitative information informs the preliminary physician referral decisions as to requirements for developmental resources. Similarly, the Bayley-III results plus the qualitative information inform the preliminary psychologist referral decisions. Both preliminary referral decisions consolidated into a brief written communication between the psychologist and the physician generally achieves consensus for final integrated decision (FID), that is communicated by the physician to the family at the end of the visit. In the case of discrepancies in decisions, these are addressed at a short meeting between the physician, psychologist and the nurse coordinator, to review the scores obtained from the ASQ3 and the Bayley-III and to discuss other qualitative information to achieve a FID between team members.

In our clinic, both the ASQ3 and the Bayley-III scores support physicians’ and psychologists’ decisions to refer to services of developmental resources including: early return to the NNFU clinic, physiotherapy; audiology and speech therapy; Ontario Early Years Center (EYC) for education on parenting skills [14]; Infant Development Program (IDP) for early developmental intervention [15]; and Ottawa Children’s Treatment Center (OCTC) [16], a local rehabilitation center, when there is a clear suspicion of developmental delay. Additional NNFU visits (early return), usually within 6 to 12 months, are suggested when the team has concerns and wants to reassess before the routine visit at 4 years of age.

### Procedures to reconstruct the decision-making process

For the purpose of this study, we conceptualized the decision making into three dimensions: the preliminary professional opinion that is based on the developmental assessment or screening tool plus the qualitative information; the theoretical referral decision (TRD) which is the referral decision based solely on the scores obtained for the developmental assessment tool (Bayley-III) or screening tool (ASQ3); and the final integrated decision (FID) which represents the interdisciplinary team referral decision made in clinic for a particular child.

In two one-hour focused ASQ-Bayley project meetings held outside of the clinical NNFU clinic hours, physicians and the psychologist working in the clinic first reviewed and created a model of how the decisions were made during the NNFU clinic visits (Fig 1); and second discussed and obtained consensus on the TRD for every possible array of test results (S1 and S2 Appendices).

To reflect the sequence in the decision-making process within our NNFU clinical practice, the study design assumed that both the physician and the psychologist independently formulate preliminary opinions at the end of their respective assessments. These preliminary opinions are based on the medical, behavioral and socio-family environmental assessment and the neurological exam (physician only), complemented by the scores obtained from the ASQ3 and Bayley-III for the physician and the psychologist respectively. Then, these preliminary opinions made by the physician (physician preliminary opinion) and made by the psychologist (psychologist preliminary opinion) are shared and inform the FID (Fig 1).

To isolate testing scores from other sources of information and to estimate how the individual scores in each tool (ASQ3 and Bayley-III) influence the clinician’s judgement and preliminary opinion, we created algorithms that represent TRDs for referrals to developmental resources. These algorithms were used solely for this research project, and are not used in clinic. The TRD algorithms captured consensus between participants of the ASQ-Bayley project meetings to predict a referral TRD corresponding to all possible hypothetical combinations of ASQ3 subscale scores based on three cut off values as per ASQ3 design (ASQ-TRD) and Bayley-III subscale scores also based on three cut-off values as per scaled score Bayley-III design (Bayley-TRD). The cut-off values based on the ASQ3 and Bayley-III for TRDs are included in S1 and S2 Appendices. For example, if a score is lower than 1 standard deviation (SD) the norm in communication on the ASQ3 or in expressive or receptive language on the Bayley-III and the other subscales are normal, a referral to audiology and speech therapy is made. Similarly for a score more than 1 SD below the norm in gross motor on the ASQ3 or on the Bayley-III, a physiotherapist should see the child, etc. After the algorithms were created, the 18-month ASQ3 scores and the Bayley-III scores were extracted from the medical charts and interpreted using a computer program created with R version 3.1 software [17]. The R program applied the cut-off ranges for normal, grey zone, and abnormal to the observed ASQ3 and Bayley-III scores. TRDs were obtained by matching these ranges to the algorithmic logic tables. The TRDs were then compared to FIDs to obtain estimates of sensitivity and specificity. The results of the computed TRDs were manually verified for each child.

### Statistical analyses

Demographic characteristics of children included in the study and those excluded for missing data were summarized using descriptive statistics. To test for demographic characteristics, differences between the excluded for missing data and included groups, Pearson’s chi-square or Fisher’s exact test and Student’s t-test were applied as appropriate. Holm’s adjustment was used for multiple testing [18]. For each participant we compared the degree of agreement according to Byrt’s definition [19] between the three levels of decisions—ASQ-TRD, Bayley-TRD and FID—by calculating kappa values for referral to each developmental resource.

To render kappa values clinically meaningful and to assess whether the ASQ3 was sufficient to inform FID decisions, we compared the three individual decisions—the ASQ-TRD, Bayley-TRD and FID—with referrals to each developmental resource.

Every chart reviewed was determined to fit into one of four clinically possible case-scenarios for decisions. The results were classified in case-scenario 1 when ASQ-TRD, Bayley-TRD and FID were all in agreement, in case-scenario 2 when only the ASQ-TRD and FID were in agreement, in case-scenario 3 when only the Bayley-TRD and FID were in agreement and finally in case-scenario 4 when neither the ASQ-TRD or the Bayley-TRD agreed with the FID, irrespective of an agreement between the ASQ-TRD and the Bayley-TRD. McNemar's test was performed to compare the proportion of ASQ-TRD results that corresponded with the FID to the proportion of Bayley-TRD results that corresponded with the FID, taking into account the pairing of the tests for each FID. To evaluate the uncertainty around the estimated difference in performance of the two tests, a 95% paired Wilson score confidence interval was computed.

Finally, following the methodology of Schonhaut [12], we computed Pearson’s correlation coefficient between the ASQ3 and Bayley-III as well as the validity properties of ASQ3 scores ≤ - 2SD to predict a score ≤ - 1SD on the Bayley-III in at least one domain.

## Results

### Study population

Of 114 charts initially screened based on gestational age less than 29 weeks discharged from the NICU, 14 severely handicapped infants had been transferred to the rehabilitation centre prior to their 18 months old visit for further in depth assessment and proper treatment, so were not seen in our clinic. Nine infants were followed in another pediatric clinic that does not use the ASQ3. We reviewed 91 charts, and after removing those with incomplete information in the medical chart (n = 24), we included 67 children who were born extremely preterm in our analyses. Reasons for incomplete information included: Bayley-III (n = 13) and/or ASQ3 (n = 2) assessment interrupted due to infant fatigue or becoming uncooperative; ASQ3 cancelled by team members’ choice after Bayley-III assessment completed for avoiding duplication of assessment (n = 6); and Bayley-III cancelled by parents after ASQ3 and physician assessment completed (n = 1). As well, one family moved out of the region, one child was sick and one family cancelled their appointment. There was no significant difference between the demographic characteristics of the infants and caregivers listed in Table 1 for children withdrawn for missing ASQ3 or Bayley-III scores, and included children.

**Table 1:** Demographic characteristics.

Characteristics*	Withdrawn for Missing Data (n = 24)	Included Population (n = 67)
Gestational age, weeks^+days^; mean (SD)	27^+3^ (1^+2^)	27^+0^ (1^+2^)
Birth weight, g; mean (SD)	1001 (218)	957(226)
Male	50.0%	49.3%
Apgar 5 min; mean (SD)	7.1 (2.1)	7.2 (2.0)
IVH grade 3 OR 4 OR PVL cystic	9.1%	9.0%
BPD	38.1%	40.3%
PDA ligation	9.1%	14.9%
Maternal age; mean (SD)	26.6 (6.1)	31.5 (4.9)
Main caregiver:		
biological mother	86.7%	92.4%
did not complete high school	0%	1.6%
completed high school	28.6%	22.6%
completed college	57.2%	56.5%
post-graduate training	7.1%	16.1%
level of education unknown	7.1%	3.2%
ASQ3 scores: mean (SD) [Range]		
Communication		39.5 (13.6) [10, 60]
Gross motor		52.6 (12.2) [5, 60]
Fine motor		51.5 (7.7) [30, 60]
Problem Solving		44.8 (9.9) [10, 60]
Personal Social		51.3 (8.6) [30, 60]
Bayley-III composite scores: mean (SD) [Range]		
Cognitive		99.6 (12.3) [55, 135]
Language		96.9 (15.9) [53, 138]
Motor		93.4 (9.5) [64, 118]

* None significant adjusted p-value for all characteristics

BPD, Bronchopulmonary dysplasia; IVH, Intraventricular haemorrhage; PDA, Patent ductus arteriosus; SD, standard deviation.

### Kappa comparisons of assessments

Kappa values comparing ASQ-TRD and Bayley-TRD ranged from poor to fair agreement (Table 2). There was fair agreement for PT, OCTC, early return and Audiology and Speech therapy. There was poor agreement for the EYC and the IDP.

**Table 2:** Agreement among screening tools for referral to developmental services—kappa coefficients.

Kappa Value (n = 67)	Early Return	Audiology & Speech Therapy	PT	IDP	OCTC	EYC	None
ASQ—TRD & BAYLEY-TRD	0.50	0.43	0.58	0.07	0.51	0.039	0.43
ASQ-TRD & FID	0.35	0.36	0.64	0.12	1.00	0.21	0.36
BAYLEY-TRD & FID	0.24	0.24	0.41	0.20	0.51	-0.05	0.33

ASQ, Ages and Stages Questionnaire; BAYLEY, Bayley Scales of Infant Development; EYC, Early Years Centre; FID, Final integrated decision; IDP, Infant Developmental Program; OCTC, Ottawa Children Treatment Centre; PT, Physiotherapy; TRD, Theoretical referral decision

For the ASQ-TRD and FID analyses, agreement ranged from poor to excellent. There was excellent agreement for OCTC, and good agreement for PT. There was a slight agreement for Audiology and Speech therapy, early return and for EYC and the agreement was poor for IDP.

The degree of agreement between the Bayley TRD and FID ranged from no agreement to fair agreement at best. There was fair agreement for OCTC and PT. For Early return, Audiology and Speech therapy there was slight agreement. The agreement was poor for IDP and none for EYC.

### Decision-making analyses

Table 3 demonstrates that for the majority of decisions (73.3%) the ASQ-TRD, Bayley-TRD and FID were all in agreement. In an additional 10% the FID was in agreement with the ASQ-TRD over the Bayley-TRD, which is comparable to the percentage of scenarios that found the FID in agreement with the Bayley-TRD over the ASQ-TRD (7.7%). Nine percent of FIDs were guided by other factors such as qualitative information, as the FID reflected neither the ASQ-TRD nor the Bayley-TRD. The McNemar test did not show a significant difference in the proportion of ASQ results that agreed with the FID (83.3%, Table 3) and the proportion of Bayley results that agreed with the FID (81.1%; p = 0.34). The difference in proportions was 2.2% (95% CI [-1.9%, 6.4%]).

**Table 3:** Utility of the assessment tools according to case-scenario.

		Final Integrated Decisions							
Case scenario	For each developmental resource	Early Return	Audiology & Speech Therapist	PT	IDP	OCTC	EYC	Total Referrals	Total%
1	ASQ TRD = BAYLEY TRD	44	34	57	47	62	51	295	**73.3%**
	ASQ TRD = FID								
	BAYLEY TRD = FID								
2	ASQ TRD ≠ BAYLEY TRD	8	10	6	6	5	5	40	**10.0%**
	ASQ TRD = FID								
	BAYLEY TRD ≠ FID								
3	ASQ TRD ≠ BAYLEY TRD	5	6	1	10	0	9	31	**7.7%**
	ASQ TRD ≠ FID								
	BAYLEY TRD = FID								
4	ASQ TRD = or ≠ BAYLEY TRD	10	17	3	4	0	2	36	**9.0%**
	ASQ TRD ≠ FID								
	BAYLEY TRD ≠ FID								
Total		67	67	67	67	67	67	402	

ASQ, Ages and Stages Questionnaire; BAYLEY Bayley Scales of Infant Development; EYC, Early Years Centre; FID, Final integrated decision; IDP, Infant Developmental Program; OCTC, Ottawa Children Treatment Centre; PT, Physiotherapy; TRD, Theoretical referral decision

Compared with Bayley-III as a gold standard to support referral decisions, we can estimate possible over- or under-referrals (Table 4) should the ASQ3 be relied upon. Based on the TRDs for each tool used, 6/67 (9.0%) of infants may have been over-referred (ASQ3 scores indicate theoretically to refer and Bayley-III not) and 12/67 (17.9%) would have been under-referred (ASQ3 scores indicate theoretically not to refer and the Bayley-III to refer).

**Table 4:** Distribution of the theoretical referral decisions based on the ASQ3 and Bayley-III scores.

		**Theoretical Referral Decisions based on Bayley-III scores**	
		Not to Refer	Refer
**Theoretical Referral Decisions based on ASQ3 scores**	Not to Refer	20 (29.9%)	12 (17.9%)
	Refer	6 (9.0%)	29 (43.3%)

ASQ: Ages and Stages Questionnaire; Bayley: Bayley Scales of Infant Development.

### Concurrent validity analyses

Pearson’s correlation between the ASQ3 and Bayley-III was 0.63. This yielded a 47% sensitivity and 94% specificity. The negative and positive predictive values were 86% and 70% respectively.

## Discussion

ASQ3 and Bayley-III scores aligned with most of the multidisciplinary team final medical decisions for referrals to developmental resources, when assessing 18 months corrected gestational age children born prematurely, at less than 29 weeks gestational age. We did not find a statistically significant difference in the performance of the ASQ3 (83.3%) compared to the Bayley (81.1%) in relation to the FID. Furthermore, the 95% confidence interval indicated that the difference between the performances of the two tests is at most 6.4% (the upper end of the 95% confidence interval). In our context, the ASQ3 in conjunction with the qualitative information seemed sufficient to guide the majority (92.3%) of the final decisions. A total of 7.7% of decisions were made based on the Bayley-III over the ASQ3 results. Proficiency of the ASQ3 is seen most clearly for children with the most critical developmental delays such as global developmental delay or cerebral palsy requiring referral to the rehabilitation center (OCTC). The ASQ3 scores seemed sufficient to guide the multidisciplinary team referral decisions, with ASQ3 kappa score (k = 1.00) for OCTC referrals, and general proficiency in all situations.

The findings should be taken with caution as many potential biases could have influenced the results. Limitations include the retrospective study design, the small sample size and the missing data that could have introduced bias. That said, the studied population was quite homogeneous (severely handicapped infants were excluded), and there was no significant difference in the demographic characteristics between the 24 infants withdrawn for missing scores and the 67 infants included in the analysis.

Another limitation is that TRDs identified retrospectively may be subject to biases that could affect the validity of our analysis. The decision process was reconstructed based on consensus among NNFU team members to reflect our clinical practice and TRDs. The FID is a documented yes/no referral extracted directly from the chart, subject to limited bias. The study does not address the use or weighting of the items of information utilized to arrive at the FID. It reflects the clinical judgment of the multidisciplinary team that made the final decisions based on each clinician’s interpretation of the ASQ3 and Bayley-III scores in association with the qualitative information that they collected. The validity of the FID is assumed as it integrates the opinions of at least two persons, supported by multiple sources of information. Our referral practice bias in favor of early intervention could have influenced the final decisions for referrals and may explain the slightly stronger kappa agreements between the ASQ-TRD and the FID compared to the Bayley-TRD and the FID. These kappa results could be an indication of over-referrals but may also be explained by the Bayley-III overestimating child development and not detecting those who needed to be referred [20,21].

Controversies about criteria for appropriate referrals are frequently raised in the literature [4]. Our multidisciplinary team members believe that referral to developmental resources should be made early when delays in milestone acquisition are identified and we usually don't refer when children are normal in all developmental domains. This is reflected in our TRD algorithms. This attitude could support the validity of our FID in which the results closely reflect the team decision making in our specific context, with the caveat that the generalizability could be limited to similar clinical practice and decision making processes. The study was not designed to determine whether the FID was correct, nor to determine the rate of over- or under-referrals. In an attempt to estimate these possibilities, we identified a concerning potential maximal risk for under-referrals up to 18% if only the ASQ3 scores are used to guide decisions compared to using the Bayley-III scores as the gold standard to support referral decisions. This risk for under-referrals could be lower in practice, particularly with consideration of other qualitative information including the medical, behavioral and neurological examinations.

It is possible that the algorithmic TRD logic tables created to determine the ASQ3 and Bayley-III TRDs do not reflect the decision-making process with exactitude. To minimize this bias, prior to any chart review we obtained group consensus among the health care providers for the TRD logic tables based on theoretical ASQ3 and Bayley-III scores, to accurately reflect the influence of the ASQ3 and the Bayley-III scores on the multidisciplinary decision-making that occurs in our NNFU clinical practice.

Measurement biases could potentially affect the results of the ASQ3 and the Bayley-III assessment. The more flexible administration procedure for the ASQ3 compared to the very rigid procedure for the Bayley-III could have influenced the scores. Both tests were administered on the same day, with the Bayley-III being scheduled before the ASQ3 for the majority of the appointments. The ASQ3 scores could be overestimations since the infant was exposed to similar exercises with the Bayley-III; conversely, the ASQ3 scores may be lower as a result of the child being fatigued from the Bayley-III. Additionally, the physicians involved in the NNFU clinic were not strictly blinded to the ASQ3 scores obtained during the previous NNFU clinic visits and to the psychologist opinion on the performance of the child doing the Bayley-III prior to administering the ASQ3. Potential influence over ASQ3 scores is mitigated by the fact that the physician did not directly observe the Bayley-III assessment and was not aware of which items the infant passed or failed. If the parents and the physician were in some cases aware of the Bayley-III performance this would be another potential confounder in our study that could inflate the correlation between the two tests and decisions. For example, parents may claim that they have seen their child performing a similar milestone during the Bayley-III assessment as was assessed with the ASQ3. Conversely, the poor to fair agreement between the ASQ-TRD and Bayley-TRD argues against the inadvertent influence of the Bayley-III results on the ASQ3 assessment. Face-to-face observations using the ASQ3 as a guide instead of asking parents to complete the scores themselves could have also helped to reduce observer bias and to improve consistency of testing and interpretation. The rationale of the face-to-face observation of the child’s performance was to increase the validity and the reliability of the physician’s clinical judgment of the child development, given that parental stress associated with the high risk status for developmental delays of their infant may influence self-reporting of the developmental concerns [22]. The non-standard use of the ASQ3 by health care professionals to guide observations during assessments is supported by the inter-observer reliability agreement over 90% between professional examiners and parents on the classification of the child’s performance [7,23]. The ASQ3 is designed to be answered by parents, therefore to ensure validity and reliability of the final ASQ3 scores, the physician conducted the testing in the presence of the parents and included their opinions on the ability of their child to perform the item assessed [23].

Based on concurrent validity psychometric properties, the current literature remains inconclusive as to whether the ASQ can be used alone to screen high-risk infants and children for developmental delays. Skellern et al. concluded that the high negative predictive value (98%) supported the use of the ASQ, but that it needed to be associated with other assessment strategies in order to identify developmental concerns in children who had been born at less than 31 weeks [9]. Woodward et al., however, concluded that the ASQ 2^nd^ edition should not be substituted for the use of the Bayley-II when assessing very low birth weight infants at 18 to 22 months corrected gestational age [10]. More recently, Simard et al. also recommended the use of additional measures to increase the proficiency to detect developmental delays based on their results looking at the concurrent validity of the ASQ and the Bayley [11]. A Chilean group concluded that the ASQ3 could be recommended for developmental screening for extremely premature infants <32 weeks gestational age given its sensitivity and specificity of 86%, and correlations with the Bayley-III scores (r = 0.65) [12]. Our analysis of the influence of the information obtained from the ASQ3 and the Bayley-III scores on clinicians’ decision-making potentially adds complementary information to the classical psychometric validity analyses when deciding which tool to use to assess children who were born very preterm. Although there are many potential drawbacks in the methods, this study allowed us to better understand biases to consider in the design of a high quality prospective study exploring the use of assessment and screening tool to support decision making to appropriately refer infants to developmental resources.

In view of the retrospective design and the potential biases, clear recommendations for NNFU practice cannot be drawn. Nevertheless, the results of this study reassured to a certain extent our local NNFU team members in their practice. To avoid duplication of the assessments and considering the costs associated with administering and interpreting the Bayley-III, it was felt that the ASQ3 done by the physician during the visit would be sufficient to support most of our NNFU clinic team decisions. The Bayley-III is still provided, however, for children born at less than 26 weeks gestational age, mainly for research purposes and to collect local standardized long term outcome statistics, to provide accurate information to mothers at risk of delivering an extremely preterm infant and their partners, when participating in a shared decision making process [24].

## Conclusion

In this retrospective study, referral to developmental resources of a very high-risk cohort of preterm infants at 18 months corrected gestational age was found to be largely congruent with findings of the ASQ3 screening tool. A complementary but novel approach to the evaluation of children at high risk of developmental delays entails the use of the ASQ3 and the Bayley-III to support referral decisions of a NNFU multidisciplinary team. Despite biases inherent in the study design, distinct from classical validity analysis, it appears that the ASQ3 along with other sources of information could potentially guide decisions when assessing development of very preterm infants in a NNFU clinic setting. The decision making process using developmental screening and assessment tools must be further explored to determine accurately the risks for over- and especially under-referral. Well-designed, larger prospective studies could investigate ways to improve developmental assessment and use of limited resources.

## Supporting information

S1 Appendix Bayley-III logic table for theoretical referral decisions. (DOCX)

S2 Appendix ASQ-III logic table for theoretical referral decisions. (DOCX)

S3 Appendix Dataset to replicate the study. (CSV)

## Abbreviations

ASQ3: Ages and Stages Questionnaire 3^rd^ Edition
Bayley-III: Bayley Scales of Infant Development 3^rd^ Edition
CHEO: Children’s Hospital of Eastern Ontario
EYC: early years center
FID: final integrated decision
IDP: infant development program
NNFU: neonatal follow up
OCTC: Ottawa children’s treatment center
SD: standard deviation
TRD: theoretical referral decision
