# Supplementary material for: ASQ3 and/or the Bayley-III to support clinicians' decision making
Source: PLoS One. 2017 Feb 2;12(2):e0170171. doi: 10.1371/journal.pone.0170171 (PMC5289417; doi:10.1371/journal.pone.0170171)
Supplement: S1 Appendix — (DOCX) [file pone.0170171.s001.docx]

| Appendix 1: Bayley-III Logic Tables | | | | | | | | | | | | |
| --- | --- | --- | --- | --- | --- | --- | --- | --- | --- | --- | --- | --- |
| Bayley-III results | | | | | | **Theoretical Decision** | | | | | | |
| Combination | Cognitive Composite | Communication | | Fine Motor Scaled Score | Gross Motor Scaled Score | Early Return | Audiology & Speech Therapy | Physiotherapy | Infant Development Program | OCTC | | Early Years Center |
|  |  | Receptive Scaled Score | Expressive Scaled Score |  |  |  |  |  |  |  |  |  |
| 1 | N | N | N | N | N |  |  |  |  |  | |  |
| 2 | A | N | N | N | N | X |  |  | X |  | | X |
| 3 | G | N | N | N | N | X |  |  |  |  | |  |
| 4 | N | A | N | N | N |  | X |  |  |  | |  |
| 5 | A | A | N | N | N | X | X |  | X |  | | X |
| 6 | A or G | A or G | N | N | N | x | x |  | x |  | | x |
| 7 | N | G | N | N | N |  | X |  |  |  | |  |
| 8 | N | N | A | N | N |  | X |  |  |  | |  |
| 9 | A or G | N | A | N | N | X | X |  | X |  | | X |
| 10 | N | A | A | N | N |  | X |  |  |  | |  |
| 11 | A or G | A or G | A | N | N | X | X |  | X |  | | X |
| 12 | N | G | A | N | N |  | X |  |  |  | |  |
| 13 | N | N | G | N | N |  | X |  |  |  | |  |
| 16 | A or G | A or G or N | G | N | N | X | X |  | X |  | | X |
| 17 | N | A | G | N | N |  | X |  |  |  | |  |
| 18 | N | G | G | N | N |  | X |  |  |  | |  |
| 19 | N | N | N | A | N | X |  |  | X |  | |  |
| 20 | A | N | N | A | N | X |  |  | X |  | | X |
| 21 | G | N | N | A | N | X |  |  | X |  | | X |
| 22 | N | A | N | A | N | X | X |  | X |  | |  |
| 23 | A or G | A | N | A | N |  |  |  |  | X | |  |
| 24 | N | G | N | A | N |  | X |  | X |  | |  |
| 25 | A or G | G | N | A | N |  |  |  |  | X | |  |
| 26 | N | N | A | A | N | X | X |  | X |  | |  |
| 27 | A or G | N | A | A | N |  |  |  |  | X | |  |
| 28 | N | A | A | A | N | X | X |  | X |  | |  |
| 29 | A | A | A | A | N |  |  |  |  | X | |  |
| 30 | G | A | A | A | N |  |  |  |  | X | |  |
| 31 | N | G | A | A | N | X | X |  | X | |  |  |
| 32 | A or G | G | A | A | N |  |  |  |  | | X |  |
| 33 | N | N | G | A | N | X | X |  | X | |  |  |
| 34 | A or G | N | G | A | N |  |  |  |  | | X |  |
| 35 | N | A | G | A | N | X | X |  | X | |  |  |
| 36 | A or G | A | G | A | N |  |  |  |  | | X |  |
| 37 | N | G | G | A | N | X | X |  | X | |  |  |
| 38 | A or G | G | G | A | N |  |  |  |  | | X |  |
| 39 | N | N | N | G | N |  |  |  | X | |  |  |
| 40 | A or G | N | N | G | N | X |  |  | X | |  | X |
| 41 | N | A | N | G | N | X | X |  | X | |  |  |
| 42 | A or G | A | N | G | N |  |  |  |  | | X |  |
| 43 | N | G | N | G | N | X | X |  | X | |  |  |
| 44 | A | G | N | G | N |  |  |  |  | | X |  |
| 45 | G | G | N | G | N | X | X |  | X | |  | X |
| 46 | N | N | A | G | N | X | X |  | X | |  |  |
| 47 | A or G | N | A | G | N |  |  |  |  | | X |  |
| 48 | N | A | A | G | N | X | X |  | X | |  |  |
| 49 | A | A | A | G | N |  |  |  |  | | X |  |
| 50 | G | A | A | G | N | X | X |  | X | |  | X |
| 51 | N | G | A | G | N | X | X |  | X | |  |  |
| 52 | A or G | G | A | G | N |  |  |  |  | | X |  |
| 53 | N | N | G | G | N | X | X |  | X | |  |  |
| 54 | A | N | G | G | N |  |  |  |  | | X |  |
| 55 | G | N | G | G | N | X | X |  | X | |  | X |
| 56 | N | A | G | G | N | X | X |  | X | |  |  |
| 57 | A or G | A | G | G | N |  |  |  |  | | X |  |
| 58 | N | G | G | G | N | X | X |  | X | |  |  |
| 59 | A or G | G | G | G | N |  |  |  |  | | X |  |
| 60 | N | N | N | N | A | X |  | X |  | |  |  |
| 61 | A | N | N | N | A | X |  | X | X | |  | X |
| 62 | G | N | N | N | A | X |  | X | X | |  |  |
| 63 | N | A | N | N | A | X | X | X |  | |  |  |
| 64 | A | A | N | N | A |  |  |  |  | | X |  |
| 65 | N | G | N | N | A | X | X | X |  | |  |  |
| 66 | A | G | N | N | A |  |  |  |  | | X |  |
| 67 | G | G | N | N | A | X | X | X | X | |  | X |
| 68 | G | G | A | N | A |  |  |  |  | | X |  |
| 69 | N | N | G | N | A | X | X | X |  | |  |  |
| 70 | A or G | N | G | N | A |  |  |  |  | | X |  |
| 71 | N | A | G | N | A | X | X | X |  | |  |  |
| 72 | A or G | A | G | N | A |  |  |  |  | | X |  |
| 73 | N | G | G | N | A | X | X | X |  | |  |  |
| 74 | A or G | G | G | N | A |  |  |  |  | | X |  |
| 75 | N | N | N | A | A | X |  | X | X | |  |  |
| 76 | A | N | N | A | A |  |  |  |  | | X |  |
| 77 | G | N | N | A | A | X |  | X | X | |  | X |
| 78 | N | A | N | A | A | X | X | X |  | |  |  |
| 79 | A or G | A or G or N | N | A | A |  |  |  |  | | X |  |
| 80 | G | A | N | A | A |  |  |  |  | | x |  |
| 81 | N | G | N | A | A | X | X | X | X | |  |  |
| 82 | N | N | A | A | A | X | X | X | X | |  |  |
| 83 | N or A or G | A or G | A | A | A |  |  |  |  | | X |  |
| 84 | N | N | G | A | A | X | X | X | X | |  |  |
| 85 | A or G | N | G | A | A |  |  |  |  | | X |  |
| 86 | N | A | G | A | A |  |  |  |  | | X |  |
| 87 | A or G | A | G | A | A |  |  |  |  | | X |  |
| 88 | N | G | G | A | A | X | X | X | X | |  |  |
| 89 | A or G | G | G | A | A |  |  |  |  | | X |  |
| 90 | N | N | N | G | A | X |  | X | X | |  |  |
| 91 | A or G | N | N | G | A | X |  | X | X | |  | X |
| 92 | N | A | N | G | A |  |  |  |  | | X |  |
| 93 | A or G | A | N | G | A |  |  |  |  | | X |  |
| 94 | N | G | N | G | A | X | X | X | X | |  |  |
| 95 | A or G | G | N | G | A |  |  |  |  | | X |  |
| 96 | N | N | A | G | A | X | X | X | X | |  |  |
| 97 | A or G | N | A | G | A |  |  |  |  | | X |  |
| 98 | N | A | A | G | A |  |  |  |  | | X |  |
| 99 | A or G | A or G | A | G | A |  |  |  |  | | X |  |
| 100 | N | G | A | G | A |  |  |  |  | | X |  |
| 101 | N | N | G | G | A | X | X | X | X | |  |  |
| 102 | A or G | N | G | G | A |  |  |  |  | | X |  |
| 103 | N or A or G | A or G | G | G | A |  |  |  |  | | X |  |
| 104 | N | N | N | N | G | X |  | X |  | |  |  |
| 105 | A or G | N | N | N | G | X |  | X | X | |  | X |
| 106 | N | A | N | N | G | X | X | X |  | |  |  |
| 107 | A or G | A | N | N | G |  |  |  |  | | X |  |
| 108 | N | G | N | N | G | X | X | X |  | |  |  |
| 109 | A | G | N | N | G |  |  |  |  | | X |  |
| 110 | G | G | N | N | G | X | X | X | X | |  | X |
| 111 | N | N | G | N | G | X | X | X |  | |  |  |
| 112 | A | N | G | N | G |  |  |  |  | | X |  |
| 113 | G | N | G | N | G | X | X | X | X | |  | X |
| 114 | N | A | G | N | G | X | X | X |  | |  |  |
| 115 | A or G | A | G | N | G |  |  |  |  | | X |  |
| 116 | N | G | G | N | G | X | X | X |  | |  |  |
| 117 | A or G | G | G | N | G |  |  |  |  | | X |  |
| 118 | N | N | N | A | G | X |  | X | X | |  |  |
| 119 | A | N | N | A | G |  |  |  |  | | X |  |
| 120 | G | N | N | A | G | X |  | X | X | |  | X |
| 121 | N | A | N | A | G | X | X | X | X | |  |  |
| 122 | A or G | A | N | A | G |  |  |  |  | | X |  |
| 123 | N | G | N | A | G | X | X | X | X | |  |  |
| 124 | A | G | N | A | G |  |  |  |  | | X |  |
| 125 | G | G | N | A | G |  |  |  |  | | X |  |
| 126 | N | N | A | A | G | X | X | X | X | |  |  |
| 127 | A or G | N | A | A | G |  |  |  |  | | X |  |
| 128 | N or A or G | A or G | A | A | G |  |  |  |  | | X |  |
| 129 | N | N | G | A | G | X | X | X | X | |  |  |
| 130 | A or G | N | G | A | G |  |  |  |  | | X |  |
| 131 | N or A or G | A | G | A | G |  |  |  |  | | X |  |
| 132 | N | G | G | A | G | X | X | X | X | |  |  |
| 133 | A or G | G | G | A | G |  |  |  |  | | X |  |
| 134 | N | N | N | G | G | X |  | X | X | |  |  |
| 135 | A or G | N | N | G | G | X |  | X | X | |  | X |
| 136 | N | A | N | G | G | X | X | X | X | |  |  |
| 137 | A or G | A | N | G | G |  |  |  |  | | x |  |
| 138 | N | G | N | G | G | X | X | X | X | |  |  |
| 139 | A or G | G | N | G | G |  |  |  |  | | X |  |
| 140 | N | N | A | G | G | X | X | X | X | |  |  |
| 141 | A or G | N | A | G | G |  |  |  |  | | X |  |
| 142 | N or A or G | A or G | A | G | G |  |  |  |  | | X |  |
| 143 | N | N | G | G | G | X | X | X | X | |  |  |
| 144 | A or G | N | G | G | G |  |  |  |  | | X |  |
| 145 | N | A | G | G | G |  |  |  |  | | X |  |
| 146 | A or G | A | G | G | G |  |  |  |  | | X |  |
| 147 | N | G | G | G | G | X | X | X | X | |  |  |
| 148 | A or G | G | G | G | G |  |  |  |  | | X |  |
|  | | | | | | | | | | | | |

| **Cognitive Composite** |
| --- |
| N: Normal = >86 |
| G: Grey = 81-85 |
| A: Abnormal =< 80 |
|  |
| **Communication Receptive Scaled Score Communication Expressive Scaled Score Fine Motor Scaled Score**  **Gross Motor Scaled Score** |
| N: Normal = >8 |
| A: Abnormal = <6 |
| G: Grey = 7 |
