# Supplementary material for: ASQ3 and/or the Bayley-III to support clinicians' decision making
Source: PLoS One. 2017 Feb 2;12(2):e0170171. doi: 10.1371/journal.pone.0170171 (PMC5289417; doi:10.1371/journal.pone.0170171)
Supplement: S2 Appendix — (DOCX) [file pone.0170171.s002.docx]

| ASQ-III Logic Table for Theoretical Referral Decisions | | | | | | | | | | | | |
| --- | --- | --- | --- | --- | --- | --- | --- | --- | --- | --- | --- | --- |
| ASQ-III results | | | | | | | **Theoretical Referral Decision** | | | | | |
| Communication | Gross Motor | | Fine Motor | Problem Solving | Personal Social | | Early Return | Audiology Speech Therapy | PT | IDP | OCTC | Early Years Center |
| Grey | Normal | | Normal | Normal | Normal | |  | X |  |  |  |  |
| Abnormal | Normal | | Normal | Normal | Normal | |  | X |  |  |  |  |
| Normal | Grey | | Normal | Normal | Normal | | X |  | X |  |  |  |
| Normal | Abnormal | | Normal | Normal | Normal | | X |  | X |  |  |  |
| Normal | Normal | | Grey | Normal | Normal | | X |  |  | X |  | X |
| Normal | Normal | | Abnormal | Normal | Normal | | X |  |  | X |  | X |
| Normal | Normal | | Normal | Grey | Normal | | X |  |  | X |  | X |
| Normal | Normal | | Normal | Abnormal | Normal | | X |  |  | X |  | X |
| Normal | Normal | | Normal | Normal | Grey | | X |  |  | X |  | X |
| Normal | Normal | | Normal | Normal | Abnormal | | X |  |  | X |  | X |
| Abnormal or Grey | Abnormal or Grey | | Normal | Normal | Normal | | X | X | X |  |  |  |
| Abnormal or Grey | Normal | | Abnormal or Grey | Normal | Normal | | X | X |  | X |  | X |
| Abnormal or Grey | Normal | | Normal | Abnormal or Grey | Normal | | X | X |  | X |  | X |
| Abnormal | Normal | | Normal | Normal | Abnormal | |  |  |  |  | X |  |
| Abnormal | Normal | | Normal | Normal | Grey | | X | X |  | X |  | X |
| Grey | Normal | | Normal | Normal | Abnormal | | X | X |  | X |  | X |
| Grey | Normal | | Normal | Normal | Grey | | X | X |  | X |  | X |
| Normal | Abnormal | | Abnormal | Normal | Normal | |  |  |  |  | X |  |
| Normal | Grey | | Grey | Normal | Normal | | X |  | X | X |  | X |
| Normal | Abnormal | | Grey | Normal | Normal | | X |  | X | X |  | X |
| Normal | Grey | | Abnormal | Normal | Normal | | X |  | X | X |  | X |
| Normal | Abnormal or Grey | | Normal | Abnormal or Grey | Normal | | X |  | X | X |  | X |
| Normal | Abnormal or Grey | | Normal | Normal | Abnormal or Grey | | X |  | X | X |  | X |
| Normal | Normal | | Abnormal or Grey | Abnormal or Grey | Normal | | X |  | X | X |  | X |
| Normal | Normal | | Abnormal or Grey | Normal | Abnormal or Grey | | X |  | X | X |  | X |
| Normal | Normal | | Normal | Abnormal or Grey | Abnormal or Grey | | X |  | x | X |  | X |
| Abnormal | Abnormal | | Abnormal | Normal | Normal | |  |  |  |  | X |  |
| Abnormal | Abnormal | | Normal | Abnormal | Normal | |  |  |  |  | X |  |
| ASQ-III results | | | | | | | **Theoretical Referral Decision** | | | | | |
| Communication | Gross Motor | | Fine Motor | Problem Solving | Personal Social | | Early Return | Audiology  Speech Therapy | PT | IDP | OCTC | Early Years Center |
| Abnormal | Abnormal | | Normal | Normal | Abnormal | |  |  |  |  | X |  |
| Abnormal | Normal | | Abnormal | Abnormal | Normal | |  |  |  |  | X |  |
| Abnormal | Normal | | Normal | Abnormal | Abnormal | |  |  |  |  | X |  |
| Normal | Abnormal | | Abnormal | Abnormal | Normal | |  |  |  |  | X |  |
| Normal | Abnormal | | Normal | Abnormal | Abnormal | |  |  |  |  | X |  |
| Normal | Normal | | Abnormal | Abnormal | Abnormal | |  |  |  |  | X |  |
| Normal | Abnormal | | Abnormal | Normal | Abnormal | |  |  |  |  | X |  |
| Abnormal | Normal | | Abnormal | Normal | Abnormal | |  |  |  |  | X |  |
| Abnormal | Abnormal | | Abnormal | Abnormal | Normal | |  |  |  |  | X |  |
| Abnormal | Normal | | Abnormal | Abnormal | Abnormal | |  |  |  |  | X |  |
| Abnormal | Abnormal | | Normal | Abnormal | Abnormal | |  |  |  |  | X |  |
| Abnormal | Abnormal | | Abnormal | Normal | Abnormal | |  |  |  |  | X |  |
| Normal | Abnormal | | Abnormal | Abnormal | Abnormal | |  |  |  |  | X |  |
| Abnormal | Abnormal | | Abnormal | Abnormal | Abnormal | |  |  |  |  | X |  |
| Normal | Grey | | Grey | Grey | Normal | | X |  |  | X |  | X |
| Normal | Grey | | Normal | Grey | Grey | | X |  |  | X |  | X |
| Normal | Grey | | Grey | Normal | Grey | | X |  |  | X |  | X |
| Normal | Normal | | Grey | Grey | Grey | | X |  |  | X |  | X |
| Grey | Grey | | Grey | Normal | Normal | | X | X |  | X |  |  |
| Grey | Grey | | Normal | Grey | Normal | | X | X |  | X |  | X |
| Grey | Grey | | Normal | Normal | Grey | | X | X |  | X |  | X |
| Grey | Normal | | Grey | Grey | Normal | | X | X |  | X |  | X |
| Grey | Normal | | Normal | Grey | Grey | | X | X |  | X |  | X |
| Grey | Normal | | Grey | Normal | Grey | | X | X |  | X |  | X |
| Special Case: Any three or more that are grey or Abnormal with at least one being Abnormal will be referred to OCTC | | | | | | | | | | | | |
| ASQ-III cut-off scores for: | | | | | | PT: Physiotherapy  IDP: Infant Developmental Program  OCTC: Ottawa Children Treatment Center (Rehabilitation center) | | | | | | |
| Communication:  Gross Motor:  Fine Motor:  Problem Solving:  Personal Social: | | Normal = 35-60; Grey = 15-30; Abnormal = 0-10  Normal = 50-60; Grey = 40-45; Abnormal = 0-35  Normal = 45-60, Grey = 35-40, Abnormal = 0-30  Normal = 40-60, Grey = 30-35, Abnormal = 0-25  Normal = 40-60, Grey = 30-35, Abnormal = 0-25 | | | |  |  |  |  |  |  |  |
